# Analysis of NHEJ-Based DNA Repair after CRISPR-Mediated DNA Cleavage

**DOI:** 10.3390/ijms22126397

**Published:** 2021-06-15

**Authors:** Beomjong Song, Soyeon Yang, Gue-Ho Hwang, Jihyeon Yu, Sangsu Bae

**Affiliations:** 1 Department of Chemistry, Hanyang University, Seoul 04763, Korea; antares119@gmail.com (B.S.); stellayang91@gmail.com (S.Y.); iamleohwang@gmail.com (G.-H.H.); muner8146@gmail.com (J.Y.); 2 Research Institute for Convergence of Basic Sciences, Hanyang University, Seoul 04763, Korea

**Keywords:** CRISPR, DNA double-strand break, non-homologous end joining, NHEJ-based DNA repair, repair accuracy, genome editing

## Abstract

Genome editing using CRISPR-Cas9 nucleases is based on the repair of the DNA double-strand break (DSB). In eukaryotic cells, DSBs are rejoined through homology-directed repair (HDR), non-homologous end joining (NHEJ) or microhomology-mediated end joining (MMEJ) pathways. Among these, it is thought that the NHEJ pathway is dominant and occurs throughout a cell cycle. NHEJ-based DSB repair is known to be error-prone; however, there are few studies that delve into it deeply in endogenous genes. Here, we quantify the degree of NHEJ-based DSB repair accuracy (termed NHEJ accuracy) in human-originated cells by incorporating exogenous DNA oligonucleotides. Through an analysis of joined sequences between the exogenous DNA and the endogenous target after DSBs occur, we determined that the average value of NHEJ accuracy is approximately 75% in maximum in HEK 293T cells. In a deep analysis, we found that NHEJ accuracy is sequence-dependent and the value at the DSB end proximal to a protospacer adjacent motif (PAM) is relatively lower than that at the DSB end distal to the PAM. In addition, we observed a negative correlation between the insertion mutation ratio and the degree of NHEJ accuracy. Our findings would broaden the understanding of Cas9-mediated genome editing.

## 1. Introduction

The field of genome editing has grown rapidly as a result of the harnessing of programmable nucleases such as zinc-finger nucleases (ZFNs) [1], transcription activator-like effector nucleases (TALENs) [2], and clustered regularly interspaced short palindromic repeat (CRISPR)-CRISPR-associated (Cas) endonucleases [3,4,5]. Until now, the CRISPR-Cas9 nuclease has superseded others because it can target genes more easily in an RNA-guided way [3,4,5,6]. The programmable nucleases commonly generate double-strand breaks (DSBs) on the target DNA, resulting in genome editing through a cell’s repair system involving a homology-directed repair (HDR), a non-homologous end joining (NHEJ) and the Ku protein-independent non-canonical NHEJ pathway such as microhomology-mediated end joining (MMEJ) [7,8,9,10,11]. In contrast with the HDR pathway that is highly faithful and error-free, the NHEJ pathway is error-prone, and small nucleotide insertions and deletions (indels) occur frequently at the cleavage site [12]. 

Despite the high fidelity, HDR-based gene editing has limitations; it is restricted to the S and G2 phases of the cell cycle [13] and thereby limited in non-dividing cells. In addition, HDR typically has low editing efficiency especially in most post-mitotic cells [14,15,16]. As an alternative, efforts to exploit the NHEJ pathway have included the introduction of a knock-in, such as a homology-independent targeted insertion (HITI) method [17], as well as a gene knock-out. Therefore, for the proper application to genome editing, understanding the accuracy of the NHEJ-mediated repair of the DSB is important. Several previous studies investigated the accurate repair by the NHEJ-mediated pathway at the DSB site generated by the Cas9 nuclease using reporter systems [18,19,20]. However, the degree of NHEJ-based DSB repair accuracy (NHEJ accuracy, hereafter) on genomic DNA has not been studied in depth thus far.

In this study, to measure NHEJ accuracy after the generation of the DSB by Cas9 nucleases, we used an exogenous DNA oligonucleotide inserted into the DSB site to prevent repetitive cleavage of the target site by the Cas9 nucleases. By inspecting the jointed DNA sequences between the exogenous DNA oligonucleotide and genomic DNA, we could determine whether the DSB was repaired flawlessly or not. Based on the measurements, we calculated NHEJ accuracy, respectively, at the DSB end proximal to a protospacer adjacent motif (PAM) and the DSB end distal to the PAM. As a result, we found that NHEJ accuracy is associated with their target sequences and is asymmetric between the DSB end proximal and distal to the PAM. In addition, we determined that the fraction of the insertion mutation of total mutations is primarily relevant to the erroneous repair of the NHEJ-mediated DSB repair.

## 2. Results

### 2.1. Introduction of Exogenous DNA Oligonucleotide for the Measurement of NHEJ Accuracy in Human Cells

**Figure 1:**
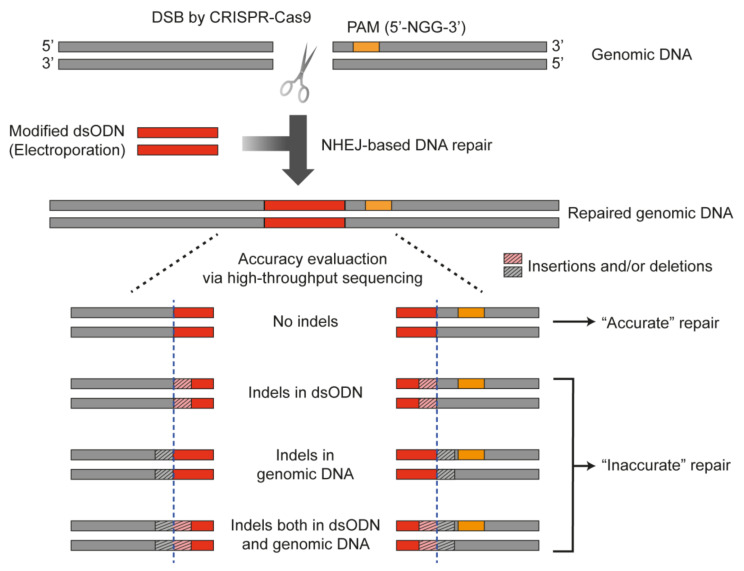
Schematic illustration of measuring the repair accuracy by the NHEJ pathway. A 34-mer oligonucleotide was used for limiting the DSB generation to one time. The accurate repair was determined only when there were no indel mutations at both the DSB and in the dsODN.

Cas9 nucleases can repeatedly cleave the target site until it is disrupted by mutations involving insertion, deletion or substitution, which might result in underestimating the degree of NHEJ accuracy. Inversely, the target site that has not been cleaved by Cas9 nucleases can be regarded as an accurate repair, which might lead to overestimating the degree of NHEJ accuracy. Thus, the evaluation of NHEJ accuracy after the generation of the DSB by Cas9 nucleases in intact cells is not easy. Modifying the target sequence after cleavage by the insertion of exogenous double-stranded oligodeoxynucleotides (dsODNs) into the DSB site is a simple way to mark the cleaved target DNA and restrict the number of cleavages by a Cas9 nuclease to one time, as in a previous study of genome-wide unbiased identifications of DSBs evaluated by sequencing (GUIDE-seq) [21]. To this end, we prepared the same dsODN, which was 34 bp in length and included a phosphorothioate modification at both 3′ and 5′ ends for the resistance to degradation by endogenous nucleases in cells. We transfected the exogenous dsODN together with other plasmids, encoding the Cas9 nuclease and single guide RNA (sgRNA) to human cells such as HeLa cells and HEK 293T cells (Figure 1). Once the exogenous dsODN was incorporated into the DSB site generated by the Cas9 nucleases, the target site could no longer be cleaved because the sgRNA could not recognize the target sequence due to the newly inserted dsODN sequences. The repaired DNA was then subjected to high-throughput sequencing (or targeted deep sequencing) for the evaluation of NHEJ accuracy on the genomic DNA. DNA alleles in which the dsODN were not incorporated were excluded from the analysis whereas DNA alleles containing dsODN were selected and used for calculating the degree of NHEJ accuracy. For the dsODN-incorporated sequences, the analyses were performed at the DSB end proximal and distal to the PAM, respectively; (i) upstream of the dsODN or relatively distal to the PAM sequence (PAM-distal, hereafter) and (ii) downstream of the dsODN or relatively proximal to the PAM (PAM-proximal, hereafter). Ultimately, DNA sequences in which there were no indels both in dsODN and genomic DNA sequences were classified as ‘accurate’ repairs whereas the others were classified as ‘inaccurate’ repairs. The NHEJ accuracy value was obtained by dividing the ‘accurate’ frequency with the total repair frequency.

### 2.2. Sequence Dependency of NHEJ Accuracy

**Figure 2:**
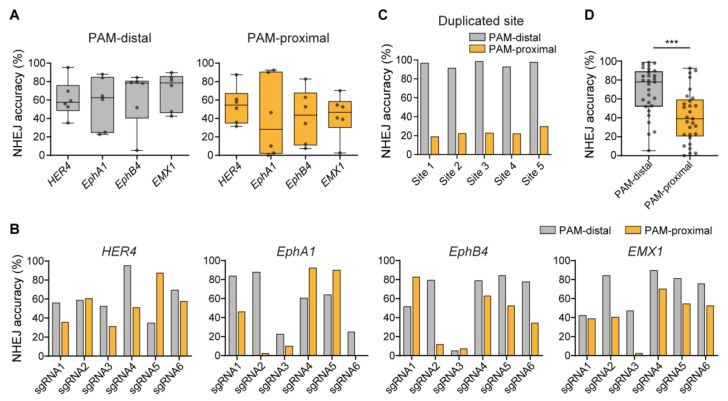
Sequence dependency of NHEJ accuracy in HeLa cells. (**A**) Comparison of NHEJ accuracy among the four genes (PAM-distal, *p* = 0.8732, Kruskal–Wallis test; PAM-proximal, F(3,20) = 0.2545, *p* = 0.8572, one-way ANOVA) (plot line: median, box: 25–75% percentiles, error bars: range). (**B**) NHEJ accuracy at each targeted site. (**C**) NHEJ accuracy at the sites having the same sequence. (**D**) Comparison of NHEJ accuracy between the PAM-distal end and the PAM-proximal end (*** *p* = 0.0007, Wilcoxon signed rank test) (plot line: median, box: 25–75% percentiles, error bars: range).

To investigate whether NHEJ accuracy was related to the sequences flanking the DSB site, we chose 24 sgRNAs (six different sites in each gene) in four genes (*HER4*, *EphA1*, *EphB4* and *EMX1*) to induce DSBs using Cas9 nucleases (Supplementary Table S1). Plasmids encoding the Cas9 nuclease and each sgRNA were co-transfected together with the exogenous dsODN to HeLa cells through electroporation. An analysis of the repair patterns was performed using high-throughput sequencing and NHEJ accuracy at the PAM-distal end and the PAM-proximal end, respectively, was calculated. According to the results, there was no significant difference in NHEJ accuracy among the genes at both the PAM-distal ends and the PAM-proximal ends, suggesting that NHEJ accuracy was independent of the gene type (Figure 2A and Supplementary Table S2). However, we found that NHEJ accuracy was variable among the six target sites within each gene (Figure 2B), suggesting that NHEJ accuracy was associated with the target sequences. To verify this finding, we additionally tested an sgRNA that could target five different endogenous sites simultaneously (i.e., the target sequence was duplicated at five different sites in the genome) (Supplementary Table S1). The results of the high-throughput sequencing revealed that NHEJ accuracy values at the five different sites were conserved regardless of the genomic position (Figure 2C), strongly supporting our hypothesis that NHEJ accuracy was governed by target sequences.

On the other hand, we found that NHEJ accuracy was quite different between the PAM-distal end and the PAM-proximal end of each targeted site, and the PAM-distal ends typically showed higher NHEJ accuracy compared with the PAM-proximal ends (Figure 2B,C). In total, a statistical analysis with 29 target sites tested revealed that the average NHEJ accuracy of the PAM-distal ends was significantly higher than that of the PAM-proximal ends (Figure 2D), suggesting that the CRISPR-Cas9 system might affect the repair differently between the two ends of the DSB.

Taken together, we concluded that (i) NHEJ accuracy is more related to the sequences flanking the DSB site rather than the gene type or the location of the DSB site and (ii) that the NHEJ-mediated DSB repair tends to be more reliable at the PAM-distal ends than at the PAM-proximal ends in general.

### 2.3. Cell Line Dependency of NHEJ Accuracy

**Figure 3:**
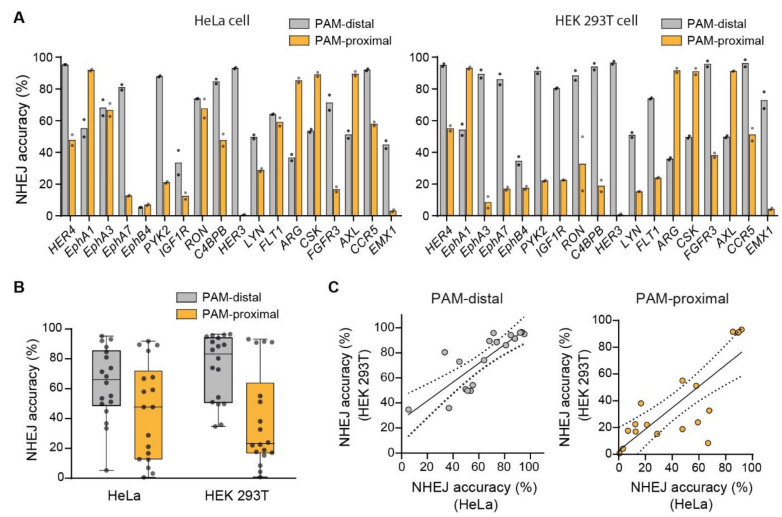
Comparison of NHEJ accuracy between HeLa and HEK 293T cell lines. (**A**) NHEJ accuracy at the PAM-distal end and the PAM-proximal end in HeLa cells and HEK 293T cells. (**B**) Comparison of NHEJ accuracy between the PAM-distal end and the PAM-proximal end in HeLa cells (PAM-distal, 63.44 ± 5.75%; PAM-proximal, 44.76 ± 7.68%; *p* = 0.07) and HEK 293T cells (PAM-distal, 74.18 ± 5.22%; PAM-proximal, 38.62 ± 7.64%; *p* < 0.005, Wilcoxon signed rank test) (plot line: median, box: 25–75% percentiles, error bars: range). (**C**) Correlation of NHEJ accuracy at each gene between HeLa cells and HEK 293T cells at the two DSB ends (PAM-distal, R^2^ = 0.8576, *p* < 0.0001, Spearman correlation; PAM-proximal, R^2^ = 0.7626, *p* = 0.0002, Spearman correlation). The linear regression fit is indicated by the solid line with 95% confidence intervals denoted by the dotted lines.

We then investigated whether the above findings were conserved across cell types. To this end, an additional 18 sgRNAs targeting different genes for DSB generation were selected (Supplementary Table S3) and tested in two different human cells, HeLa and HEK 293T cells. The high-throughput sequencing results revealed that the general features of NHEJ-mediated DSB repair were conserved in both cell lines. NHEJ accuracy was typically higher at the PAM-distal ends than at the PAM-proximal ends in most of the targeted genes in both cells although NHEJ accuracy values were variable among the genes (Figure 3A). A further statistical analysis with 18 target sites showed that the average NHEJ accuracy of the PAM-distal ends was substantially higher than that of the PAM-proximal in both HeLa cells and HEK 293T cells (Figure 3B and Supplementary Table S4). In addition, consistent with our above observation that NHEJ accuracy is related to the sequences flanking the DSB site, we found that NHEJ accuracy in each gene between the two cell types was positively correlated at both the PAM-distal end and the PAM-proximal end (Figure 3C). These results suggest that the features of the NHEJ-mediated DSB repair were conserved across cell types.

### 2.4. Correlation between NHEJ Accuracy and the NHEJ-Mediated Insertion Mutation Ratio 

**Figure 4:**
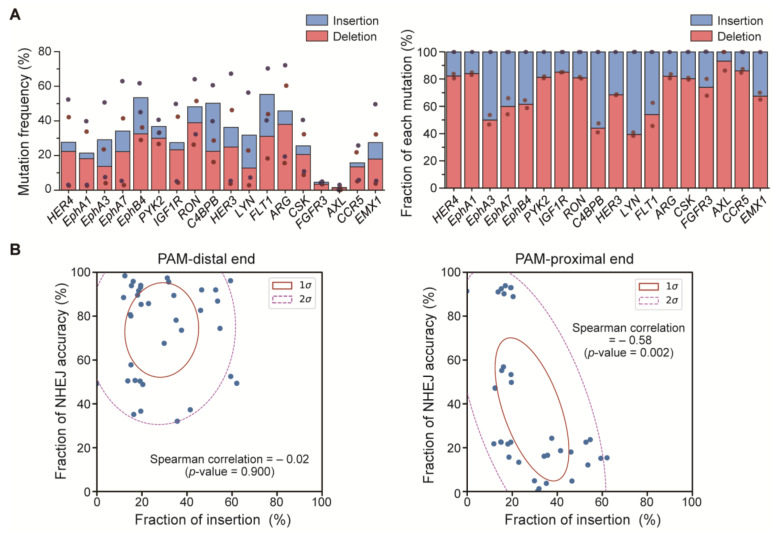
Correlation between the repair accuracy and the ratio of insertion mutation and deletion mutation. (**A**) (**Left**) The frequency of each type of mutation. (**Right**) The relative fraction of the insertion and the deletion. (**B**) Correlation between the repair accuracy and the fraction of insertion mutation in the PAM-distal end and the PAM-proximal end in HEK 293T cells (PAM-distal, R^2^ = −0.02, *p* = 0.900, Spearman correlation; PAM-proximal, R^2^ = −0.58, *p* = 0.002, Spearman correlation).

Indel mutations are frequently generated at the DSB site during the NHEJ-mediated repair process. In addition, a previous study reported that individual targets of the CRIPSR-Cas9 have distinct preferences for the type of mutation [22]. For example, Cas9-mediated gene editing outcomes in several targets contained a relatively high portion of insertion patterns compared with the deletion while other targets exhibited few insertion patterns. Therefore, we assumed that the indel profile might be correlated with the degree of NHEJ accuracy. To address this, we assessed the mutation type (insertion or deletion) in the 18 targets (used above) in HEK 293T cells. The assessment was performed in the absence of the dsODN to figure out the mutation type over the DSB site not localized to either one end of the DSB. As shown in Figure 4A, the indel profile was diverse across the 18 genes, consistent with the previous report. We then compared the fraction of insertion against NHEJ accuracy of either the PAM-distal end or the PAM-proximal end. Notably, we found a negative correlation of the fraction of the insertion mutation with NHEJ accuracy at the PAM-proximal end of the DSB whereas NHEJ accuracy at the PAM-distal end of the DSB was not governed by either the insertion or the deletion alone. (Figure 4B). This result suggested that the high fraction of the insertion mutation might be a primary cause of the inaccurate repair at the PAM-proximal end of the DSB.

### 2.5. Application of NHEJ-Mediated DSB Repair Using Dual sgRNA for Precise Large Deletion

**Figure 5:**
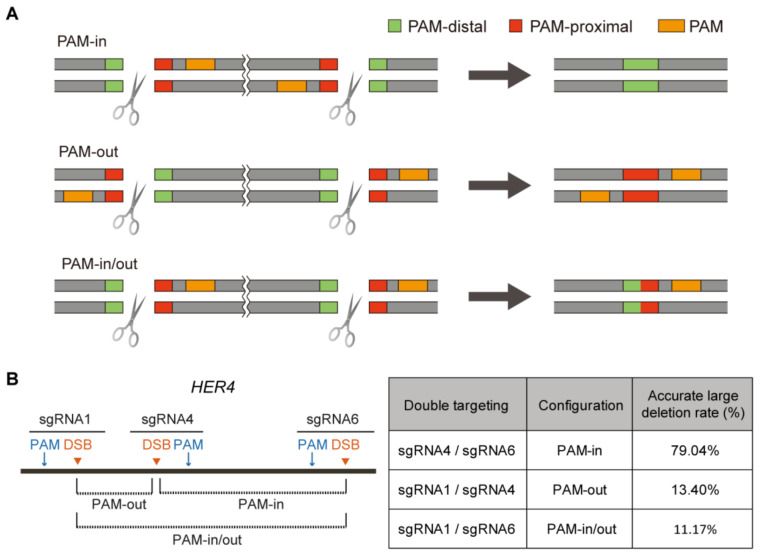
Precise DNA deletion using an NHEJ-mediated DSB repair. (**A**) Schematic illustration of three different deletion configurations. (**B**) (**Left**) The three different deletion configurations in the *Her4* gene. (**Right**) The accurate large deletion rate of each configuration.

Given our observation that the NHEJ-mediated DSB repair at the PAM-distal end was more accurate, we sought to apply this feature for precise deletion using dual sgRNA. Depending on the position of the DSB site relative to its PAM sequence, the DNA ligation could occur between two PAM-distal ends (PAM-in, hereafter), between two PAM-proximal ends (PAM-out, hereafter) or between one PAM-distal end and one PAM-proximal end (PAM-in/out, hereafter) during the DSB repair (Figure 5A). Based on the results described above, we expected that the PAM-in configuration should exhibit a higher NHEJ accuracy than the others. Consistent with our expectation, a much higher NHEJ accuracy was observed for the PAM-in compared with the PAM-out or the PAM-in/out in the *HER4* gene of HeLa cells (Figure 5B).

## 3. Discussion

In the current study, we measured and compared the accuracy of the repair by the NHEJ pathway on the DSB generated by the Cas9 nucleases in human-originated cells. To assess the accuracy of the NHEJ-mediated repair for one working event, we added an exogenous DNA oligonucleotide that could label the DSB event of the cleaved target sites and prevent repetitive DSB generation by a Cas9 nuclease. From our strategy, we observed three major findings as follows: (i) NHEJ accuracy was independent of the target location or the cell types but dependent on the sequence flanking the DSB site (the average NHEJ accuracy was 75% in maximum), (ii) the NHEJ-mediated DSB repair was typically more error-prone at the PAM-proximal end of the DSB and (iii) the fraction of insertions in the total mutations showed a negative correlation with the degree of NHEJ accuracy.

Our results suggested that two ends of the DSB site might go through differently via CRISPR-Cas9 nucleases. The release of cleaved DNA products by Cas nucleases is thought to be extremely slow [23,24,25]. Prolonged staying of the Cas nucleases may hinder access of other protein factors required for an accurate DSB repair by the NHEJ pathway, thereby inducing an erroneous repair such as indel generation. It was recently reported that the dissociation of the PAM-distal DNA from a nuclease occurs earlier than that of the PAM-proximal DNA [26,27,28] suggesting a tighter binding of the PAM-proximal DNA to a Cas nuclease. Therefore, the difference in the repair accuracy between the two DSB ends may arise from the difference in stay time of the two cleaved DNA products in a Cas9 nuclease after the cleavage. In the future, this hypothesis can be verified by comparing the results from CRISPR-Cas9 with the results from other programmable nucleases such as TALENs. As the two DSB ends bind symmetrically to each subunit in the TALEN system [2], NHEJ accuracy might be similar between both the 5′ and 3′ end of the DSB generated by TALENs.

On the other hand, other molecules other than Cas nucleases may affect the accuracy of the DSB repair by the NHEJ pathway. It has been reported that the interaction between the RNA polymerase II and proteins associated with a DSB repair such as the catalytic subunit of DNA protein kinase (DNAPKcs) and Ku protein [29,30,31]. Therefore, the transcriptional state of the DNA near the DSB site might affect the accuracy of the DSB repair.

Although we found a tendency that NHEJ accuracy was higher at the PAM-distal end compared with the PAM-proximal end, a few targets showed opposite results. Considering that these opposite tendencies in a few targets were conserved in both HeLa and HEK 293T cells, the results suggested that at least it was not due to the experimental variation. One possible explanation for this inconsistency is that the sequences flanking the DSB site might be important in determining NHEJ accuracy at the PAM-distal end and the PAM-proximal end. If the sequence at the PAM-distal end is vulnerable to the erroneous repair, the degree of NHEJ accuracy at the PAM-distal end could be lower than that at the PAM-proximal end. 

Another interesting finding of our study was that there was an inverse correlation between the fraction of insertions of the mutations and the degree of NHEJ accuracy at the PAM-proximal end of the DSB. This result indicated that an inaccurate repair was more frequent at the PAM-proximal end of the DSB and that it might be associated with previous observations that Cas9 nucleases frequently generate staggered ends with 5′ overhang at the cleavage site resulting in a 1 bp insertion (identical to a 4th base upstream of the PAM) mutation more abundantly [22,32,33,34,35,36,37,38]. Similar to previous studies, our data also showed that the inserted 1 bp sequences were mostly identical to a 4th base upstream of the PAM (Supplementary Figure S1 and Supplementary Table S5). As the fraction of the 1 bp insertion was diverse across the target sites, Cas9 nucleases might not always induce the staggered end but have a sequence dependency. Given our observations in this study and in previous findings, we postulated that Cas9 nucleases preferably induced the staggered end at a few target sequences and thereby the NHEJ-mediated DNA repair became more inaccurate at those targets. To prove this, a future study would include high-throughput experimental data (> 1000 targets) and a machine-learning-based analysis. 

The higher NHEJ accuracy at the PAM-distal end of the DSB site suggested the feasibility of precise DNA deletion by the generation of two DSBs. Indeed, we confirmed a much higher NHEJ accuracy in the PAM-in configuration (Figure 5). Many kinds of genetic diseases arise from DNA duplication such as Duchenne muscular dystrophy [39], Pelizaeus–Merzbacher disease [40], Charcot–Marie–Tooth [41], ataxia-telangiectasia [42], Alport syndrome [43] and MECP2 duplication syndrome [44]. Therefore, our strategy of precise DNA deletion can be a relevant method for treating such diseases. To test the potential of the precise DNA deletion as treatments of diseases, it is required to be tested in patient-derived primary cell lines bearing the erroneous genes.

In summary, our current study investigated the features of a DSB repair mediated by the NEHJ pathway and will be informative in its application in diverse ways including gene therapy.

## 4. Materials and Methods

### 4.1. Cell Culture Conditions

HeLa (CCL-2, ATCC, Rockville, MD, USA) and HEK 293T (CRL-11268, ATCC, Rockville, MD, USA) cell lines were maintained in Dulbecco’s modified eagle’s medium (DMEM) supplemented with 10% fetal bovine serum (FBS), 100 µg/mL streptomycin, 100 unit/mL penicillin and 0.1 mM non-essential amino acids.

### 4.2. sgRNA Preparation

All sgRNAs were designed using a Cas-designer to target each gene or site and included a 5′-NGG-3′ PAM sequence [45]. A pRG2-GG vector containing a U6-driven gRNA scaffold was used for sgRNA cloning. The cloning was confirmed by a restriction enzyme cut with BamH I and Hind III.

### 4.3. Transfection Conditions

A Nucleoflector 4D (Lonza, Basel, Switzerland) was used for HeLa cells and HEK 293T cells. The SpCas9 plasmid (500 ng) and the sgRNA plasmid (500 ng) were electroporated into 1.0 × 10^5^ cells according to the manufacturer’s protocol. SE solution (Lonza, Switzerland) and SF solution (Lonza, Basel, Switzerland) were used for HeLa cells and HEK 293T cells, respectively. In the presence of the dsODN, the SpCas9 plasmid (500 ng), the sgRNA plasmid (500 ng) and the dsODN (100 pM, 1 μL) were electroporated into 1.0 × 10^5^ cells using the same protocol. For the large deletion experiment, the SpCas9 plasmid (750 ng) and each sgRNA plasmid (250 ng) were transfected. The genomic DNA was isolated from the whole cell population using NucleoSpin Tissue (MACHEREY-NAGEL GmbH & Co. KG, Düren, Germany) 72 h after transfection. 

### 4.4. Targeted Deep Sequencing

The targeted sites were amplified with a Phusion HF DNA polymerase (New England BioLabs Inc., Boston, MA, USA) to produce a sequencing library. The libraries were sequenced using MiniSeq with the TruSeq HT Dual Index system and a paired-end system (Illumina, San Diego, CA, USA). The NGS results were joined using a Fastq-join tool (https://github.com/brwnj/fastq-join [46]. The insertions and deletions were confirmed using Cas-Analyzer (http://www.rgenome.net/cas-analyzer/) [47]. 

### 4.5. Accuracy Analysis

The analysis program was developed using Python 3. The analysis of the accuracy of the NHEJ pathway was performed through the following steps: (i) The Fastq-join sequence was sorted by the 12 bp indicator sequence that was 70 bp apart from the cleavage at both the PAM-distal end and the PAM-proximal end. (ii) The same read was counted and removed when the count of read was 1. (iii) If the reads had the WT marker that was ±5 bp at the cleavage sites of the reference sequence, the reads were classified as a “wild type”, which was excluded from the calculation of the repair accuracy. (iv) If the reads had 10 bp in the inserted dsODN sequence, the reads were classified as “dsODN inserted reads”. (v) The remaining reads were classified as “mutation reads”. (vi) In the dsODN inserted reads, if the reads had accurately 10 bp at the end of the dsODN and at the end of the cleavage sites, the reads were classified as an “accurate repair”. The analysis process for the accuracy of dual gRNAs was the same as (i)–(iii) of the NHEJ accuracy analysis. The accuracy was determined by the marker that was 5 bp before and after the two target cleavage sites. The script used for the analysis is available in github (https://github.com/Gue-ho/NHEJ_accuracy).

### 4.6. Statistics

Statistical tests were described in the main text. All statistical tests were two-sided (* *p* < 0.05, ** *p* < 0.01, *** *p* < 0.001). A parametric statistical test (paired *t*-test and a one-way ANOVA) was used only for the data set that passed the Shapiro–Wilk normality test. Otherwise, the Wilcoxon signed rank test and Kruskal–Wallis test were used.

## Data Availability

High-throughput sequencing and WGS data have been deposited in the NCBI Sequence Read Archive database (SRA; https://www.ncbi.nlm.nih.gov/sra (accessed on 15 June 2021)) under accession number of PRJNA731544.

## Supplementary Materials

The following are available online at https://www.mdpi.com/article/10.3390/ijms22126397/s1.
