# Supplementary material for: Analysis of NHEJ-Based DNA Repair after CRISPR-Mediated DNA Cleavage
Source: Int J Mol Sci. 2021 Jun 15;22(12):6397. doi: 10.3390/ijms22126397 (PMC8232687; doi:10.3390/ijms22126397)
Supplement: Supplementary file 1 [file ijms-22-06397-s001.zip › ijms-1253002-supplementary.pdf]

SUPPLEMENTARY MATERIALS

Analysis of NHEJ-based DNA repair after CRISPR-mediated DNA cleavage

Table of Contents

Supplementary Figure S1. The fraction of non-random 1-bp insertion.

Supplementary Table S1. Sequence of dsODN and target sites of each gene.

Supplementary Table S2. NHEJ accuracy at the two DSB ends in each gene.

Supplementary Table S3. Targeted sequence of each gene.

Supplementary Table S4. NHEJ accuracy in each gene.

Supplementary Table S5. The fraction of predictable 1-bp insertion among the total 1-bp insertion in the two human cell lines.

Supplementary Figure S1. The fraction of non-random 1-bp insertion. The fraction of 1-bp insertion which is identical to -4bp upstream of the PAM is diverse across the genes.

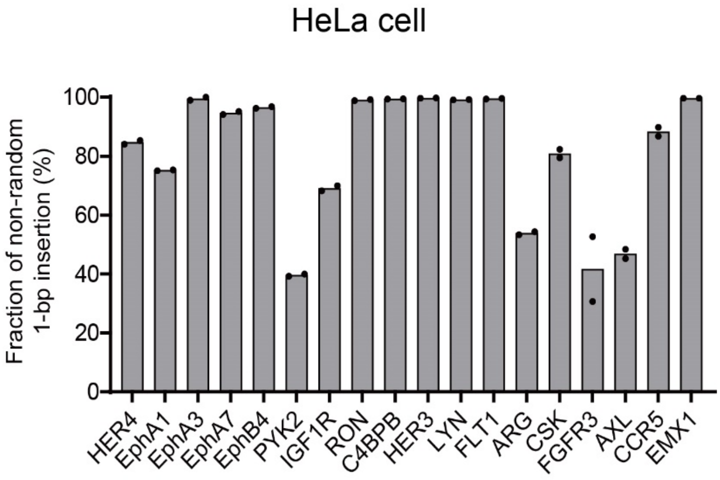

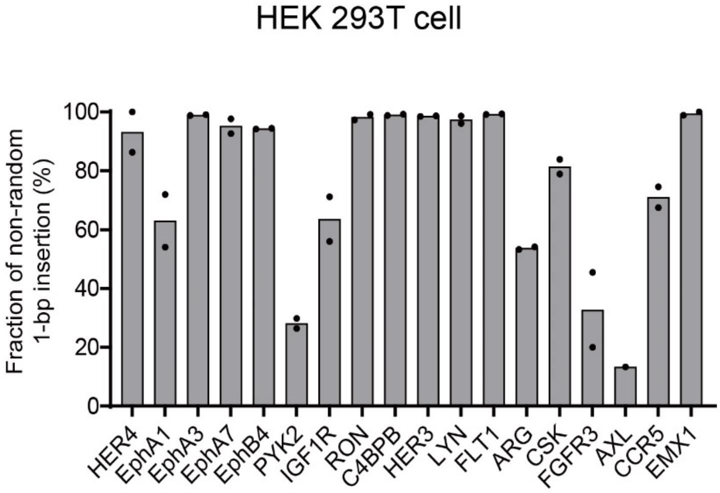

Supplementary Table S1. Sequence of dsODN and target sites of each gene.

<table>
<tr><td colspan="2">dsODN</td><td colspan="3">5'-P-G*T*TTAATTGAGTTGTCATATGTTAATAACGGT*A*T-3'
3'-C*A*AATTAACTCAACAGTATACAATTATTGCCA*T*A-P-5'</td></tr>
<tr><th colspan="2">Gene</th><th>sgRNA</th><th>Targeted sequence</th><th>Strand</th></tr>
<tr><td colspan="2" rowspan="6">HER4</td><td>1</td><td>CTTGGATTAAAGAACTTGACAGG</td><td>-</td></tr>
<tr><td>2</td><td>TTATGAGGATCGATATGCCTTGG</td><td>-</td></tr>
<tr><td>3</td><td>TCGTGGGACAAAACTTTATGAGG</td><td>-</td></tr>
<tr><td>4</td><td>ATAATGCGTAAATTCTCCAGAGG</td><td>+</td></tr>
<tr><td>5</td><td>AGTCACAGGCTACGTGTTAGTGG</td><td>-</td></tr>
<tr><td>6</td><td>CAATTCTTTAGAATATGATATGG</td><td>-</td></tr>
<tr><td colspan="2" rowspan="6">EphA1</td><td>1</td><td>AAGGTCTCCTTGCAGCCCAGAGG</td><td>+</td></tr>
<tr><td>2</td><td>GAAACTCTTGCAGTCCCGCACGG</td><td>+</td></tr>
<tr><td>3</td><td>ACTGCAGCTCCACGTGGACGCGG</td><td>+</td></tr>
<tr><td>4</td><td>GCTCCAATTGGATCTACCGCGGG</td><td>-</td></tr>
<tr><td>5</td><td>GACGCAGAGACACTGACCACTGG</td><td>-</td></tr>
<tr><td>6</td><td>TACCAGGACTGCCCAATGCAAGG</td><td>-</td></tr>
<tr><td colspan="2" rowspan="6">EphB4</td><td>1</td><td>GTGGGTTGCCCTAATTTGATGGG</td><td>-</td></tr>
<tr><td>2</td><td>AAACACGGACAGTATCTCATCGG</td><td>-</td></tr>
<tr><td>3</td><td>GCAGAATATTCGGACAAACACGG</td><td>-</td></tr>
<tr><td>4</td><td>GCTCTGCTTCCTGTAGCCGATGG</td><td>+</td></tr>
<tr><td>5</td><td>CTGAGTCACACATCTTTATGAGG</td><td>+</td></tr>
<tr><td>6</td><td>TTATGAGGCACACACACCAAAGG</td><td>+</td></tr>
<tr><td colspan="2" rowspan="6">EMX1</td><td>1</td><td>GACTGAGGCTACATAGGGTTAGG</td><td>-</td></tr>
<tr><td>2</td><td>GCCGTTTGTACTTTGTCCTCCGG</td><td>-</td></tr>
<tr><td>3</td><td>GAGTCCGAGCAGAAGAAGAAGGG</td><td>+</td></tr>
<tr><td>4</td><td>AGGGCTCCCATCACATCAACCGG</td><td>+</td></tr>
<tr><td>5</td><td>CACGAAGCAGGCCAATGGGGAGG</td><td>+</td></tr>
<tr><td>6</td><td>GTCACCTCCAATGACTAGGGTGG</td><td>+</td></tr>
<tr><td rowspan="5">Duplicated sequence</td><td>Chr3:75395213</td><td>1</td><td rowspan="5">GCAGCTCTATGGAGGCCTGGNGG</td><td>+</td></tr>
<tr><td>Chr5:28926841</td><td>2</td><td>+</td></tr>
<tr><td>Chr1:144720581</td><td>3</td><td>+</td></tr>
<tr><td>Chr13:25017428</td><td>4</td><td>+</td></tr>
<tr><td>Chr9:40883645</td><td>5</td><td>+</td></tr>
</table>

* represents phosphorothioate internucleotide linkages.

Supplementary Table S2. NHEJ accuracy at the two DSB ends in each gene.

sgRNA	*HER4*		*EphA1*		*EphB4*		*EMX1*	
	PAM-distal	PAM-proximal	PAM-distal	PAM-proximal	PAM-distal	PAM-proximal	PAM-distal	PAM-proximal
1	56.1%	35.8%	83.9%	46.3%	51.8%	82.9%	42.5%	39.1%
2	59.1%	60.8%	88.0%	2.5%	79.5%	12.0%	84.5%	40.6%
3	52.7%	31.4%	22.7%	10.2%	5.3%	7.5%	47.3%	2.6%
4	95.4%	51.2%	60.7%	92.5%	79.2%	63.0%	89.8%	70.3%
5	35.0%	87.5%	64.3%	90.0%	84.5%	52.7%	81.5%	54.7%
6	69.7%	57.8%	25.0%	0.0%	78.0%	34.6%	75.8%	52.6%
Avg. NHEJ accuracy	61.3 ±8.2%	54.1 ±8.2%	57.4 ±11.5%	40.3 ±17.5%	63.0 ±12.5%	42.1 ±12.1%	70.2 ±8.2%	43.3 ±9.4%

Supplementary Table S3. Targeted sequence of each gene.

Gene	Targeted sequence	Strand	Location
HER4	ATAATGCGTAAATTCTCCAGAGG	+	Chr2
EphA1	GCTCCAATTGGATCTACCGCGGG	-	Chr7
EphA3	TTGTCGACCAGGTTTCTACAAGG	+	Chr3
EphA7	CACCTGGTATGTTCGTATCGGGG	+	Chr6
EphB4	GCAGAATATTCGGACAAACACGG	-	Chr7
PYK2	GGTCCTGAATCGTATTCTTGGGG	+	Chr8
IGF1R	TCAGTACGCCGTTTACGTCAAGG	+	Chr15
RON	GTCATCGGGCCGGTTATGGTGGG	+	Chr3
C4BPB	AATGACCACTACATCCTCAAGGG	+	Chr1
HER3	ACCATTGCCCAACCTCCGCGTGG	+	Chr12
LYN	TTAATCATGTCGCTGATACAGGG	-	Chr8
FLT1	AAAATAAGAGAGCTTCCGTAAGG	-	Chr13
ARG	TCCATCTCGCTCAGGTACGAGGG	-	Chr1
CSK	CTGACCGACCCCTAGACCGCAGG	-	Chr15
FGFR3	CGGCAACTACACCTGCGTCGTGG	+	Chr4
AXL	GTCCCGTGTCGGAAAGCTGCAGG	-	Chr19
CCR5	TGACATCAATTATTATACATCGG	+	Chr3
EMX1	GAGTCCGAGCAGAAGAAGAAGGG	+	Chr2

Supplementary Table S4. NHEJ accuracy in each gene.

Gene	HeLa cell		HEK 293T cell	
	PAM-distal	PAM-proximal	PAM-distal	PAM-proximal
HER4	95.32%	47.75%	94.98%	55.11%
EphA1	55.22%	91.92%	54.23%	93.19%
EphA3	68.19%	66.76%	89.43%	8.47%
EphA7	81.02%	12.68%	86.04%	17.08%
EphB4	5.32%	6.97%	34.70%	17.59%
PYK2	87.99%	21.10%	91.38%	22.09%
IGF1R	33.50%	12.46%	80.40%	22.56%
RON	73.84%	67.68%	88.47%	32.74%
C4BPB	84.74%	47.69%	94.07%	18.84%
HER3	93.11%	0.65%	96.52%	0.77%
LYN	49.76%	28.78%	50.94%	15.30%
FLT1	64.04%	59.18%	74.00%	23.99%
ARG	36.73%	85.41%	35.91%	91.61%
CSK	53.58%	89.17%	49.61%	90.95%
FGFR3	71.38%	16.68%	95.79%	38.11%
AXL	51.22%	89.58%	49.82%	91.24%
CCR5	92.09%	57.98%	96.16%	51.21%
EMX1	44.86%	3.20%	72.89%	4.32%
Avg. NHEJ accuracy	63.44±5.75%	44.76±7.68%	74.18±5.22%	38.62±7.64%

Supplementary Table S5. The fraction of predictable 1-bp insertion among the total 1-bp insertion in the two human cell lines.

Gene	HeLa cell	HEK 293T cell
HER4	84.70%	93.16%
EphA1	75.21%	63.01%
EphA3	99.50%	98.95%
EphA7	94.65%	95.18%
EphB4	96.53%	94.35%
PYK2	39.59%	28.09%
IGF1R	69.07%	63.56%
RON	99.04%	98.22%
C4BPB	99.45%	99.03%
HER3	99.72%	98.63%
LYN	99.20%	97.39%
FLT1	99.52%	99.26%
ARG	53.86%	53.71%
CSK	80.88%	81.41%
FGFR3	41.65%	32.73%
AXL	46.81%	13.33%
CCR5	88.31%	71.02%
EMX1	99.68%	99.45%
